# Genomic Diagnosis for Pediatric Disorders: Revolution and Evolution

**DOI:** 10.3389/fped.2020.00373

**Published:** 2020-07-08

**Authors:** Emilie Lalonde, Stefan Rentas, Fumin Lin, Matthew C. Dulik, Cara M. Skraban, Nancy B. Spinner

**Affiliations:** ^1^Division of Genomic Diagnostics, Department of Pathology and Laboratory Medicine, School of Medicine, Children's Hospital of Philadelphia, University of Pennsylvania, Philadelphia, PA, United States; ^2^Division of Human Genetics, Department of Pediatrics, School of Medicine, Children's Hospital of Philadelphia, University of Pennsylvania, Philadelphia, PA, United States

**Keywords:** genetic syndromes, genomic diagnostics, genetics, pediatrics, sequencing, copy number variants, next-generation sequencing

## Abstract

Powerful, recent advances in technologies to analyze the genome have had a profound impact on the practice of medical genetics, both in the laboratory and in the clinic. Increasing utilization of genome-wide testing such as chromosomal microarray analysis and exome sequencing have lead a shift toward a “genotype-first” approach. Numerous techniques are now available to diagnose a particular syndrome or phenotype, and while traditional techniques remain efficient tools in certain situations, higher-throughput technologies have become the *de facto* laboratory tool for diagnosis of most conditions. However, selecting the right assay or technology is challenging, and the wrong choice may lead to prolonged time to diagnosis, or even a missed diagnosis. In this review, we will discuss current core technologies for the diagnosis of classic genetic disorders to shed light on the benefits and disadvantages of these strategies, including diagnostic efficiency, variant interpretation, and secondary findings. Finally, we review upcoming technologies posed to impart further changes in the field of genetic diagnostics as we move toward “genome-first” practice.

## Introduction

Tools for genomic diagnosis have evolved rapidly over the past two decades, resulting in remarkably improved diagnostic rates as well as a significant increase in the number of disease genes. The core technologies for genetic testing laboratories have evolved from Sanger sequencing and karyotype analysis to Next Generation Sequencing (NGS)-based tests (targeted panels, exomes, and genomes) and chromosomal microarrays (CMA) (Table 1). As a result, the scope of identifiable mutations now ranges from changes in the *amount* of a particular genomic locus, such as loss or gain of entire chromosomes (i.e., aneuploidy) or smaller regions of DNA (i.e., copy number variants, CNVs), to changes in the *structure* of the genome (i.e., translocations, inversions, insertions), and to changes in the *sequence* of the genome (i.e., single nucleotide variants and short insertions/deletions). These advances have had a profound impact on how we diagnose patients who present with clinical features of known genetic disorders. In the realm of cytogenetics, traditional techniques such as cytogenetic analysis of banded chromosomes are still warranted for some conditions (e.g., trisomies, Turner syndrome). However, classic cytogenetic deletion syndromes (such as Wolf-Hirschhorn and Cri-du-Chat syndromes) are now better tested by CMA, with the opportunity to identify smaller deletions or duplications including precise breakpoints.

**Table 1:** Genomic technologies for chromosomal and molecular syndromes.

	**Types of aberrations**	**Resolution**	**Clinical indication examples**
Karyotyping	Large structural changes: aneuploidies, translocations, isochromosomes, rings, CNVs >5–10 Mb, etc. Balanced changes (translocations, insertions, inversions, rings)	5–10 Mb* *depends on region and banding level	- Suspicion of chromosome syndrome - Infertility or recurrent miscarriage - Rule out structural variant after microarray finding
FISH	Aneuploidies, CNVs, translocations, inversions, insertions Probes must be designed for specific aberration	50 kb−1 Mb; most 200–400 kb	- Prenatal aneuploidy - Parental studies for proband with structural rearrangement (balanced or imbalanced) or CNV - Follow-up studies after abnormal karyotype (e.g., SRY FISH on abnormal Y)
SNP array	Copy number changes associated with *unbalanced* structural changes; Regions of homozygosity/Uniparental disomy; Mosaicism	10–100 kb *depends on probe density and reporting criteria may be significantly larger	- Congenital anomalies - Intellectual disability
aCGH	Gene or exon level copy number changes associated with *unbalanced* structural changes	Based on designed, clinical grade typically single-exon resolution for genes of interest	- As part of a phenotype-specific panel test - A complement to exome sequencing
MLPA, real-time PCR	Deletions or duplications	Exon-level	- SMA - Thalassemia - Imprinting disorders
NGS panel or exome	SNVs, indels, copy number changes Mitochondrial DNA if long-range PCR used first	SNVs: single-nucleotide CNVs: exon-level unless breakpoint within exon, then nucleotide-level as most panels only cover exonic regions	- Phenotype-specific gene panel
NIPS	Chromosomal aneuploidies and recurrent deletion/duplication syndromes	Variable depending on methodology. Some designed to detect recurrent CNVs	-Prenatal aneuploidy screening
Sanger sequencing	Sequence variants including SNVs, small indels; CNVs smaller than the amplicon size can also be detected but not typical usage	1 bp	- Specific phenotype known to be caused by sequence variants in a single gene - Targeted testing for familial variant
Repeat-primed PCR	Repeat expansions	Quantify 1–220 repeats; Detect up to 1,000 repeats	Repeat expansion disorders
MS-MLPA and MS-qPCR	Deletion, UPD and imprinting center defect in the imprinted regions	Exon level	Imprinting disorders such as Prader-Willi Syndrome

Advances in molecular technologies have had an even more transformative effect on diagnostics. The shift to NGS-based sequencing methods has made evaluation of larger portions of the genome possible simultaneously, resulting in targeted panels including only a few or up to hundreds of genes, as well as examination of the entire exome [i.e., the protein coding regions of the genome, see section Exome Sequencing (ES)] or genome. These improvements allow a streamlined approach to diagnosis of genetic disorders, by avoiding unnecessary evaluations and diagnostic studies such as Ophthalmology and Cardiology evaluations for hearing loss patients or functional studies for patients with Fanconi anemia or Osteogenesis Imperfecta. This has allowed faster and improved diagnostic rates of heterogeneous disorders (e.g., Osteogenesis Imperfecta, Noonan syndrome, and Cornelia de Lange syndromes). Highly heterogeneous phenotypes like hearing loss, intellectual disability, and/or seizures are now amenable to diagnosis using large, NGS-based panels. The ability to test a large number of genes (if not all) simultaneously has had a significant effect on the practice of Clinical Genetics, as it becomes possible to take a “genotype-first” approach to diagnostics.

**Figure 1:**
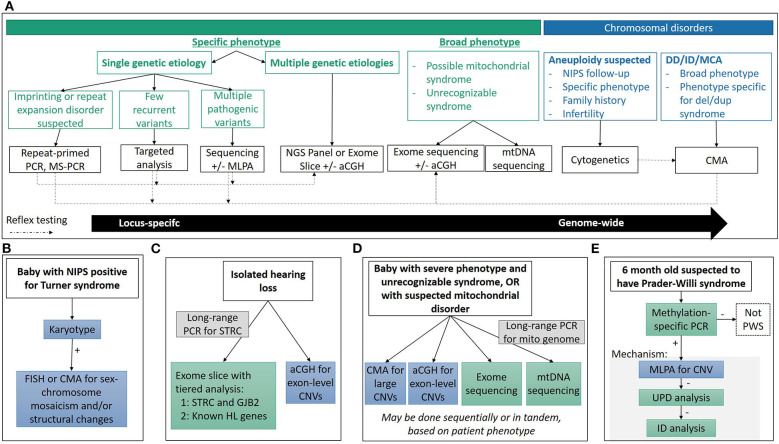
Diagnostic technologies and applications. **(A)** General considerations used in determining the appropriate technology used for diagnosis. Choosing the appropriate technique is a multi-factorial process, depending on reason for study, clinical presentation and associated genetic heterogeneity, molecular mechanisms, time and cost considerations, among others, and there is often no single “right” approach. **(B–E)** Common clinical diagnostic workflows for various genetic syndromes. See clinical examples in text for more detail.

In this review, we will discuss current core technologies for the identification of cytogenetic (section Chromosomal and Copy Number Variations), sequence (section Sequencing Variants), and other (section Disorders Requiring Special Testing) alterations causing a variety of genetic disorders (Figure 1A), and we will illustrate how these technologies have impacted our ability to make genomic diagnoses, identify new genetic causes of disease and expand the phenotypic spectrum for many classic genetic disorders (section Impact on Clinical Care). Finally, we will review several new and emerging technologies currently or predicted to further impact the field of pediatric genetic diagnostics (section Emerging Technologies).

## Chromosomal and Copy Number Variations

### Brief Description and Historical Perspective

Cytogenetic analysis has traditionally involved detection of the number and composition of metaphase chromosomes based on examination in the light microscope. However, in recent years with the development of molecular techniques capable of identifying chromosome aneuploidies and smaller copy number deletions or duplications, that is copy number variants (CNVs), the line between cytogenetic and molecular (DNA-based) genetic testing has become blurred. There now exist multiple techniques to identify changes chromosomal aneuploidies, CNVs, structural variants, or even to evaluate for absence of heterozygosity which could be related to uniparental disomy or identity by descent (Table 2).

**Table 2:** Technical comparison of technologies available for detection of chromosomal changes and/or CNVs.

**Abnormality**	**G-banding**	**FISH**	**SNP array**	**aCGH**	**MLPA**	**NGS panel or exome**	**NIPS**	**WGS**
Aneuploidy	+	+	+	+	+	+	+	+
Balanced rearrangement (translocation, inversion, insertion)	+	+	–	–	–	–	–	+
CNVs >5–10 Mb	+	+	+	+	+/–**	+	+	+
CNVs <5–10 Mb	–	+	+	+	+	+	+/–	+
Single exon deletion	–	–	–	+	+	+/–	–	+
Polyploidy	+	+	+	–	–	+	+	+
Min. mosaicism	10% with 30-count	2–5% depending on probe characteristics	5–10%*	20%*	40% for dup; 20–30% del;	Depends on coverage	Depends on methodology	Unclear
Clonal relationships	Y	Y	N	N	N	N	N	N

* *Depends on probe coverage and size of CNVs*.

** *MLPA is a targeted CNV detection strategy, and if the region of interest is involved in a much larger CNV, a CNV would be detected but additional technologies are required to delineate the size and breakpoints*.

Cytogenetic discoveries have been driven by technical innovation. The characterization of human cells having 46 chromosomes followed the discovery that chromosomes could be visualized by treating cells with colchicine (a microtubule inhibitor) and hypotonic solution (to swell the cells) (1–4). Three years later, the first chromosomal disorder was described by Lejeune in 1959, associating Down Syndrome with trisomy of “the smallest chromosome” (5), and within 1 year the genetic basis of Turner, Klinefelter, XXX, trisomy 13 and trisomy 18 were elucidated (6–10). These findings are quite remarkable given that the first *banded* human karyotype was still 10 years from being described. The development of G-banding in 1971, which is still the most commonly used banding technique today in North America, resulted in widespread adoption of cytogenetics, and numerous subsequent discoveries of chromosomal abnormalities in a constitutional and neoplastic setting (11, 12).

“Molecular cytogenetics” emerged in the 1980s with the development of fluorescence *in situ* hybridization (FISH), which determines the presence or absence of discrete segments of DNA, thus bridging molecular and cytogenetic analysis. FISH allows targeted identification of deletions and duplications associated with known disorders, such as Cri-du-Chat or Wolf-Hirschhorn syndromes, as well as characterization of translocations or marker chromosomes identified in karyotypes. The revolutionary development of PCR which, along with many other applications, allowed for targeted CNV detection of even smaller regions, and ultimately led to the development of numerous other CNV assays which rely on PCR, such as multiplex ligation-dependent probe amplification (MLPA) and chromosomal microarray analysis (CMA). Indeed, modern CMA, which enable unbiased scanning of the genome to detect CNVs, are a result of decades of work in molecular biology, bio-engineering and robotics, and have resulted in the discovery of numerous disorders associated with submicroscopic CNVs and elucidation of the underlying mechanism (Table 3) (13–18).

**Table 3:** Examples of syndromes associated with recurrent and non-recurrent CNVs. See Spinner et. al. (13) for further details including clinical descriptions.

**Syndrome**	**CNV**	**Recurrent breakpoints?**	**Notes**
1p36	1p36 deletion	No	Variable size from 0.5 to 10 Mb; ~50% due to terminal deletion, ~30% interstitial deletion, and remainder due unbalanced to rearrangements
Wolf-Hirschhorn	4p partial deletion (4p-)	No	Critical region is 4p16.3 (165 kb); ~45–50% due to unbalanced translocation
Cri-du-chat	5p partial deletion (5p-)	No	Critical regions: - Cat-like cry: 1.5 Mb region of 5p15.31 - Speech delay: 3 Mb region of 5p15.33-5p15.32. - Dysmorphic facial features: 2.4 Mb region of 5p15.31-p15.2 ~90% associated with *de novo* deletions
Williams	7q11.23 deletion	Yes	1.5 Mb deletion involving 25 genes in >90% patients Critical genes: - *ELN*: cardiovascular and connective tissue phenotypes - *LIMK1*: impaired visual motor integration - *BAZ1B*: hypercalcemia
Miller-Dieker	17p13.3 deletion	No	*LIS1* is responsible for lissencephaly, and mutations in *LIS1* result in isolated lissencephaly
Hereditary neuropathy and pressure palsies (HNPP)	17p12 deletion	Yes	1.5 Mb deletion in 80% of patients *PMP22* is critical gene
Charcot Marie Tooth Type 1	17p12 duplication	Yes	1.5 Mb duplication, reciprocal to HNPP deletion *PMP22* is critical gene
Smith-Magenis	17p11.2 deletion	Yes	3.7 Mb deletion in >90% patients *RAI1* associated with sleep disturbances
Potocki-Lupski	17p11.2 duplication	Yes	3.7 Mb duplication, reciprocal to Smith-Magenis deletion
22q11.2	22q11.2 deletion	Yes	3.0 Mb deletion in 85%, rest have deletions associated with two of four recurrent breakpoints >90 genes involved, *TBX1* may be partially responsible for cardiac abnormalities

Today, chromosome analysis using conventional cytogenetic techniques remains a first tier test for patients with a suspected aneuploidy (trisomies or sex chromosome aneuploidy), family history of a rearrangement (translocation or inversion), or infertility (19). However, for more subtle changes, such as deletions or duplications, CMA has proven to have increased sensitivity, and is the first tier test for patients with developmental delay, intellectual disability, autism spectrum disorder, or multiple congenital anomalies of unknown etiology (19). Furthermore, development of next-generation sequencing (NGS) has provided the ability to simultaneously (i.e., with a single assay) detect chromosomal aneuploidies, CNVs and sequence variants. There is thus another shift in the field as laboratories are starting to rely more on NGS for CNV detection, and less on CMA or targeted approaches.

This shift in technologies toward NGS, coupled with the ability to identify fetal DNA in maternal serum, has greatly impacted prenatal aneuploidy screening with the implementation of non-invasive prenatal screening (NIPS) (20–22). As NIPS providers are starting to offer screening for microdeletion syndromes, genome-wide aneuploidy detection (23) and even single gene disorders (24, 25), we anticipate a shift in diagnosis from the phenotypic-driven postnatal setting to screening-based prenatal detection.

### Technologies

Multiple assays can detect chromosomal aberrations (Table 2), and thus understanding the differences in their clinical utility and limitations is important (Table 1). The following sections describe and contrast chromosomal and CNV technologies as they are used today.

#### Cytogenetics (Karyotype and FISH)

In general, karyotype analysis is indicated if one suspects a chromosomal aneuploidy or a large (>5–10 Mb) structural change, especially if balanced. Only karyotype analysis, whole genome sequencing, and FISH (if the abnormality is known a priori) can detect balanced rearrangements but whole genome sequencing is currently cost-prohibitive for most indications. Molecular cytogenetics using FISH tends to be used in familial studies, cytogenetic follow-up studies, or to test for recurrent changes, such as William's syndrome (see caveats in clinical examples below). Because karyotype and FISH analysis involve visualization of individual cells, clonal relationships can be resolved when multiple cell lines are present (26). For example, a patient with a mosaic gain found by CMA requires cytogenetic studies to determine the exact number of copies (i.e., duplication, triplication, etc.), the location of the additional material (i.e., tandem duplication or inserted elsewhere), and the orientation of the additional material (i.e., inverted or not).

#### Chromosomal Microarray Analysis (CMA)

There are two major type of arrays to detect CNVs, genome-wide single-nucleotide polymorphisms (SNP) arrays which assay SNPs across the genome, and array comparative genomic hybridization (aCGH) which use larger oligonucleotide probes tiled across the genome, often with a “backbone” of SNP probes. SNPs are generally benign, single-base DNA changes that are present at an appreciable frequency in normal populations. SNP probes can detect deviations in the allele ratio which can help identify low-level mosaicism, and are required to identify regions of homozygosity due to identity by descent or uniparental isodisomy with CMA. Both arrays are widely used and have overlapping clinical utility but SNP arrays are best used to identify multi-gene or larger CNVs, identify regions of homozygosity, and can better detect low level mosaicism, while aCGH is particularly useful for gene-level CNVs, with some clinical aCGH platforms designed to identify CNVs as small as one exon (27–29). This distinction is due to the underlying technology with aCGH oligonucleotide probes capable of capturing any unique sequence in the genome while SNP array probes are limited to specific positions in the genome known to be polymorphic, which tend to fall outside coding regions. The exact resolution of both types of arrays depends on the designed probe density.

The resolution of SNP arrays is typically in the range of 10–100 kb for a clinical platform. This drastic increase in resolution compared to karyotype analysis allows for the detection of small CNVs including those found at translocation breakpoints, and elucidation of gene content of CNVs. In practice, clinical laboratories have larger size thresholds for reporting CNVs, typically between 200 kb and 1 Mb, but may choose to report smaller CNVs with clear clinical significance (19). Balanced changes such as inversions, insertions or balanced translocations cannot be detected since there is no net loss in material and no visualization of chromosomes. Similarly, when a duplication is detected, its orientation or placement within the genome cannot be deduced. Identification of CNVs of unknown significance or CNVs associated with disorders outside of the clinical indication pose significant challenges in CMA interpretation. These issues are discussed in relation to sequence variants below (section Next Generation Sequencing).

#### Multiple Ligation-Dependent Probe Amplification (MLPA)

MLPA is a semi-quantitative technique to assess copy number via multiplexed fragment size analysis (30, 31). Sequence-specific probe pairs are hybridized to the DNA of interest, ligated and amplified, and each target region has a “stuffer sequence” of a specific length allowing for identification of the multiplexed targets. MLPA is best suited for detection of smaller CNVs, such as single gene deletions or recurrent microdeletion/duplication syndromes. Commercial kits from MRC-Holland (www.mlpa.com/) are available for loci commonly involved in CNVs, such as *DMD* associated with Duchene Muscular Dystrophy (see below) and *HBB* associated with Beta-thalassemia, among many others.

#### NGS Panels and Exomes

In recent years, NGS has been widely adopted in clinical diagnostic laboratories due to its ability to efficiently interrogate multiple genes at a time. Additionally, NGS offers the ability to detect both sequence variants and structural variants (including deletions, duplications, inversions, translocations, and other alterations). For targeted panels evaluating coding regions only, structural variant detection is limited to exonic CNVs, and breakpoints typically cannot be identified. Standardized methods are not yet available and laboratories need to develop and validate internal bioinformatics pipelines, and characterize the pipeline's performance metrics in terms of minimum size of detection and mosaicism. While clinical laboratories have reported very high analytical sensitivity of NGS-based CNV detection, analytical specificity is lower particularly for smaller CNVs (1–3 exons) which typically require orthogonal confirmation (32–35). However, the major advantage with calling CNVs using NGS data is the decrease in cost compared to running two different assays (i.e., sequencing and MLPA or aCGH). Additional details of NGS are described in section Next Generation Sequencing.

#### Non-invasive Prenatal Screening (NIPS)

The ability to assay fetal cells in maternal serum has greatly altered the practice of prenatal diagnostics (20–22). It is beyond the scope of this article to fully review detailed methodological and clinical characteristics of NIPS, but readers are encouraged to refer to existing reviews [e.g., (36, 37)]. Prenatal screening for aneuploidies such as Trisomies 13, 18, and 21, which have a combined incidence of 4 in 1,000 newborns and are associated with advanced maternal age, is offered to all pregnant women using various strategies (38). In the traditional approach, a woman is offered a maternal serum screening test which measures specific maternal hormones and fetal metabolites, and women flagged as “high-risk” for fetal aneuploidy are subsequently offered a diagnostic test, either CVS or amniocentesis to collect placental tissue or fetal cells, respectively, which is sent for karyotype or FISH analysis. Now with NIPS, maternal plasma, which contains circulating fetal and maternal DNA, is analyzed with NGS or SNP arrays, as early as 10 weeks gestation. The major advantage of NIPS compared to previous serum screening is the decrease in false positive rate (or increase in specificity) without compromising the sensitivity, thus reduction of unnecessary invasive procedures (CVS or amniocentesis).

However, it must be stressed that NIPS is a *screening* test and findings from NIPS must be confirmed by a diagnostic method using cytogenetic techniques. Several reports of misuse and misunderstanding of NIPS include mis-interpretation of findings as diagnostic, or using NIPS to screen for genetic conditions other than those indicated such as sequencing changes (21, 39). False positives and false negatives are rare but do occur at a clinically significant rate due to both biological and technical reasons, including confined placental mosaicism, maternal aneuploidies or CNVs, occult maternal malignancy, vanished twin and true fetal mosaicism (40, 41). The positive predictive value for NIPS in women of advanced maternal age is highest for trisomy 21 (>90%), but decreases below 90% for trisomy 13 and 18, and as low as ~50% for sex chromosomal aneuploidies (42, 43). Because the risk of aneuploidy increases with maternal age, the positive predictive value is even lower for younger women.

### Clinical Examples

#### Turner Syndrome: Multiple Roads to Diagnosis—Karyotype, FISH, CMA, and NIPS

Turner syndrome (45,X) occurs in ~1 in every 2,500 live births, and accounts for 6–8% of spontaneous abortions (11). If Turner syndrome is suspected, karyotype analysis is the recommended technique, with some important subtleties (Figure 1B). Only 45% of Turner syndrome cases are due to a 45,X karyotype, while the rest have structurally abnormal X or Y chromosomes, and/or mosaicism for another cell line with a different sex complement (XX, XXX, XY, etc.) which may have phenotypic and/or management consequences including predisposition to gonadoblastoma if Y chromosome material is present (11, 44). Therefore, when a karyotype consistent with Turner syndrome is identified, an appropriate work up must rule out the presence of Y chromosome material, as well as characterization of any identified abnormal sex or marker chromosomes by FISH or CMA. While it may be tempting to order a CMA as the first-tier test, karyotype analysis is needed to identify structural rearrangements involving the sex chromosomes.

Sex chromosomal aneuploidies are commonly screened for in NIPS, but as discussed above, the positive predictive value is low, especially for Turner syndrome. This is at least partially due to maternal mosaic aneuploidies, such as age-related (benign) loss of X or undetected XXX syndrome, which can result both in false positive and false negatives in relation to the fetus (45). Thus, one should be wary of NIPS results that have not been confirmed by a diagnostic method, and if clinically indicated, order cytogenetic confirmatory studies. This situation may become more common as women are declining prenatal diagnostic/confirmation studies and there is ambiguity in responsibility and possible lack of communication between prenatal obstetrical care and postnatal pediatric care (42).

#### Potocki-Lupski and Smith-Magenis: CMA vs. FISH

The development of CMA led to the discovery of multiple syndromes caused by submicroscopic deletions or duplications, including so-called genomic syndromes which are caused by misalignment of repetitive sequences during recombination (16). The resulting deletions and duplications are recurrent with the same breakpoints identified in most patients, and deletions and duplications result in different, and in some cases, mirror phenotypes (Table 3).

One example of reciprocal deletion/duplication syndromes is the Smith-Magenis syndrome, which is most commonly caused by a 3.6 Mb deletion of 17p11.2 including the *RAI1* gene, and Potocki-Lupski syndrome caused by a duplication of the same region (46–49). While both syndromes are characterized by intellectual disability and sleep and behavior disturbances, they differ in many other respects and are clearly two distinct pathological entities (50). FISH studies can be used to detect these CNVs, and indeed were once the preferred test, but CMA is now the most appropriate assay due to the ability to define the deletion size and gene content, to detect atypical CNVs, and to interrogate CNVs at other loci which cover a broader differential diagnosis (48, 49, 51). FISH studies remain useful for follow-up parental studies in patients with typical CNVs.

#### Duchenne Muscular Dystrophy: Detection of Exon-Level CNVs With MLPA

For some conditions with very specific phenotypes and a high rate of deletion or duplications, MLPA is considered a first-tier test as it offers a fast and sensitive approach to molecular diagnosis. For instance, in Duchenne muscular dystrophy 65–80% of patients have a deletion or duplication within the *DMD* gene located on the X chromosome (52). High-resolution characterization of these CNVs is critical for patient management, as individuals with out-of-frame CNVs tend to present with the more aggressive phenotype of Duchenne muscular dystrophy, while those with in-frame CNVs will present with the milder Becker muscular dystrophy. Together with the characteristic presentation of a boy with progressive proximal weakness and high serum creatine phosphokinase values, a targeted MLPA panel is appropriate for molecular diagnosis in that it is rapid, cost-efficient and most importantly can determine the frame of CNVs. While aCGH platforms *may* have the resolution to detect single exon changes, SNP arrays most likely do not, and overall CMA is not recommended for molecular diagnosis of muscular dystrophy (52).

## Sequencing Variants

### Brief Description and Historical Perspective

DNA-based molecular diagnostics originated following the introduction of techniques for manipulation and analysis of DNA including the discovery and utilization of restriction enzymes (“molecular scissors”) in the late 1960s and early 1970s (53) and development of DNA-based hybridization methods, including the Southern blot in 1975 (54). The earliest applications of these methods were used for those few disorders whose molecular basis was known during the 1970s. Early molecular diagnostic tests included prenatal diagnosis focused on the well-studied disorders of hemoglobin including α and β thalassemia and sickle cell anemia (55–57).

Methods for DNA sequencing were also introduced during the 1970s (58, 59) with increasing automation and continuous improvements over the next four decades. The introduction of PCR in the mid 1980s, with its ability to target and amplify specific segments of DNA, revolutionized molecular biology as well as medical genetics, paving the way for analysis of the increasing number of known disease genes. The early explosion of tools for DNA analysis presented tantalizing possibilities to understand the genetic basis of disease, and then in 1991 The Human Genome Project was officially launched, with the goal of “Understanding Our Genetic Inheritance” by sequencing all of the bases in the human genome and creating maps of each chromosome. The first draft of the human genome was published in Nature in 2001 (60) and the “final” draft followed 3 years later in 2004 (61). As the human genome project was progressing, several technical advances in genetic analysis were also introduced that have had wide impact on genomic diagnostics including CMA and NGS, paving the way for clinical exome sequencing (ES) and genome sequencing (GS) (19, 62–64).

A consequence of the enormous resources and the explosion in technology to analyze the human genome, has been a parallel revolution in the identification of human disease genes. In 1986 as sequencing was becoming mature and PCR was introduced, there were <10 human genetic disorders for which the disease gene was known. This number ballooned to close to 950 by 2000 as the first draft of the human genome was being finalized, to over 4,000 by 2016 driven by the utilization of genome-wide tests such as ES and GS (65). Currently, there are over 9,600 conditions with a known genetic cause that can be tested using genomic technologies (https://www.ncbi.nlm.nih.gov/gtr/).

### Methods for Genetic Testing

Genetic tests analyze chromosomes, DNA, RNA, proteins, or metabolites in order to identify alterations that are associated with a clinical disease phenotype. For purposes of this section of this review, it is assumed that we are discussing changes in DNA that lead to altered gene products including single nucleotide variations, nucleotide level insertions or deletions, and generally intragenic variants. The techniques discussed here are those most commonly used for genetic testing, and are not meant to provide an exhaustive discussion. In the early years of clinical molecular genetic diagnostics, there were a number of techniques for the identification of altered sequence that are primarily hybridization based, which have almost completely been supplanted by DNA sequencing. In this review, we will limit our discussion to techniques that are in current usage, primarily sequencing.

#### Sanger Sequencing

Sanger sequencing (chain termination method), first introduced in 1977 works using the selective incorporation of nucleotides that are modified such that they “terminate” the sequencing reaction, thereby identifying the position of the base last incorporated (59). Sanger sequencing is a mature technology with excellent accuracy and is considered the “gold standard” for sequence determination, although it does not capture mosaicism below 15–20%. Sanger sequencing is most useful for disorders caused by a single, shorter gene where PCR amplicons are sequenced with highly focused accuracy and relatively long reads (~600–800 base pairs). Note deletions, duplications or structural genome rearrangements will not be identified by this technology, and it is often necessary to include a secondary method to identify these alterations. While Sanger sequencing is being replaced in many assays by NGS-based approaches, it retains an important role in the clinical laboratory for diagnosis of single-gene disorders, as an orthogonal method to confirm sequence variants identified by NGS, and as a means to provide coverage for genomic regions that are not well-covered by NGS testing due to poor capture or amplification.

#### Next Generation Sequencing

Several papers published in 2005 described advances that became known as “next generation” or “massively parallel” sequencing. The critical advance was the ability to multiplex, such that a complex library of DNA templates could be presented to the sequencing reagents simultaneously, followed by *in vitro* amplification of each template. With Illumina sequencing chemistry, for example, sequencing then proceeds by analysis of labeled nucleotides and subsequent imaging, known as “sequencing by synthesis.” Between 2005 and 2012, improvements in sequencing were fast and furious, with increasing accuracy and throughput and decreasing costs, such that beginning around 2011, sequencing of large portions of the genome became possible in the clinical diagnostic laboratory (66, 67).

##### Targeted gene panels

The first clinical application of NGS was to capture and analyze a small groups of genes to look for disease predisposing or disease-causing variants. In 2010, this method was applied to analysis of 21 genes involved in breast and ovarian cancer, when Walsh et al., simultaneously analyzed the sequence for these genes following hybridization capture in a cohort of 20 women who had a known mutation in one of the breast and ovarian cancer predisposition genes (68). This successful proof of principle study laid the foundation for a wide variety of targeted panels that are currently in use in diagnostic laboratories around the world. Targeted panels generally analyze between 2 and 500 genes, with different diagnostic strategies depending on details and specificity of the clinical phenotype, the differential diagnosis and the genetic heterogeneity of the phenotype. The major advantages of targeted gene panels compared to ES are (1) higher depth sequencing, and (2) guaranteed 100% coverage of genes which may require Sanger fill-in.

##### Exome sequencing (ES)

Most disease-causing variants (~85%) are concentrated in the 1–2% of the genome that is protein coding (exonic regions), and the collection of all of the exons is known as the exome. These exonic regions can be selectively “captured,” allowing sequencing of these high yield genomic regions, reducing the amount of sequence significantly in comparison to the genome, allowing for a relatively efficient test for both new causes of genomic disease or identification of pathogenic variants in known disease genes (69, 70). The diagnostic yield of ES varies between 10 and over 50%, depending on the clinical features of the population tested, year of testing, and analytical strategy (66, 67, 71–73). For example, diagnostic yield of exome sequencing for chronic kidney disease is 9% based on one recent study (74), compared to 29–55% for neurodevelopmental disorders, and 26–58% in unselected cohorts with a suspected genetic etiology (75). It has greatly impacted the practice of clinical genetics, since the requirement for a precise clinical phenotype is less stringent, in contrast to the ordering of specific single-gene tests, where a decision regarding which gene to test has to be made up front. Indeed, a recent meta-analysis on the diagnostic yield of ES for neurodevelopmental disorders concluded that ES should be considered as a first line test for these patients (76) (see section Genotype-First Approach for further discussion).

### Challenges in Interpretation of NGS Data

The techniques for exome capture and sequencing have matured greatly and are relatively stable in most laboratories, but the primary challenges today are in the complexity of data analysis and interpretation of the clinical significance of variants identified. The large amount of sequence data generated by NGS platforms has fueled the addition of bioinformatics teams into clinical diagnostic laboratories to develop data handling and analysis pipelines for these complex tests. Once sequencing is completed, the sequence is aligned and compared to the reference genome to identify sequence variants and filters are applied to retain only high quality variants. Variants that are rare in the population and are predicted to have a functional impact on the gene, mostly commonly by altering the gene's protein coding sequence (non-sense, missense, frameshift mutation, etc.), are typically prioritized for analysis and human interpretation.

#### Analytical Sensitivity and Specificity

Short read sequencing by NGS is prone to both false positives and false negatives, with analytical sensitivity and specificity depending on assay design (amplification strategy, target coverage, instrumentation, etc.). False positives can occur due to PCR artifacts, sequencing errors, low coverage, and errors in alignment, among other reasons (77). False positives tend to have characteristic features which enables clinical laboratories to either filter them away, or to devise analytical standards to defining which variants require orthogonal confirmation (78). Similarly, false negatives can be due to one of several technical reasons. First, like all amplification-based technologies, variants present in the primer or probe binding sets can result in allele-drop out, where only one strand of DNA is amplified and analyzed. Next, sequence context such as high GC-content (e.g., in promoter regions) and repetitive and complex genomic regions (e.g., pseudogenes) can result in lower coverage and misalignment, respectively. To help laboratories handle these and other related challenges, several professional groups have developed standards and guidelines for the implementation, bioinformatics and analysis of next-generation sequencing assays, including strategies for handling repetitive regions and low-coverage regions (79, 80). Finally, because laboratories restrict analysis to variants most likely to impact the protein sequence, non-coding, intronic and synonymous variants which could impact the transcription of genes may be overlooked. Indeed, coupling genome and transcriptome assays (i.e., DNA and RNA analysis) has shown promise in increasing the clinical sensitivity of diagnostic testing (see section RNA-Sequencing and Methylation Pattern Analysis) (81).

#### Variant Classification

Beyond analytical considerations, a significant remaining challenge is the interpretation of the clinical significance of the variants identified. A variant is assessed to determine the likelihood that the it results in a non-functional or poorly functioning protein. This requires consideration of the inheritance of the variant, the literature detailing the genes functions, the expression profile in human tissues, the mechanism by which mutations are known to cause specific phenotypes (haploinsufficiency, dominant negative), frequency of the particular variant in the general population using databases such as gnomAD (82) and frequency of the variant in patients with clinical abnormalities using the general literature and databases such as ClinVar (83). These latter steps are straightforward in principle but difficult in practice, and variant interpretation has been the subject of a number of Guideline and Recommendation papers from the American College of Medical Genetics and Genomics and the Association for Molecular Pathology (79, 84, 85). Indeed, even among highly experienced laboratories, there is often disagreement between the classification of a variant as Pathogenic, Likely Pathogenic, Uncertain [Variant of Uncertain Significance (VUS)], Likely Benign or Benign. Further, high-throughput sequencing can reveal potentially pathogenic variants in genes with limited clinical validity, that is, genes with insufficient information regarding their association with a genetic syndrome or phenotype. As a result, such variants are also classified as VUS. Reporting of variants of uncertain significance can be difficult for clinicians to interpret, and this is an area of active work to develop methods to lessen the uncertainty in interpretation.

#### Incidental and Secondary Findings

The ability to look for alterations in a large percentage of human genes in one test has many obvious advantages over the tedious process of examining genes one at a time. However, it is now well-understood that in addition to finding pathogenic variants that might explain a patient's clinical problems, there is the possibility of finding a wide variety of variants unrelated to this phenotype. Identification of medically actionable findings has become a critical part of genome wide tests such as ES. The medical genetics community has worked diligently to develop guidelines for providing medically actionable incidental findings to patients undergoing NGS based testing. The ACMG has adopted a new term, “secondary findings,” to refer to pathogenic variants in a list of 59 genes that are deemed “medically actionable.” Clinical laboratories are recommended to specifically look for pathogenic variants in these 59 genes (therefore not incidental), and report them if patients would like to have this information provided (86). Significantly, it is recommended that VUS's in these genes not be reported, as it is felt that these would cause more confusion than benefit (87). It is important to contrast secondary findings from incidental findings, which are pathogenic variants in genes unrelated to a patients' clinical indication and which are not curated as a secondary finding gene by the ACMG; reporting of these variants are laboratory-specific. For further discussion of how secondary findings impact pediatric care, see section Counseling and Ethical Considerations.

### Clinical Examples

#### B-Globin: Sanger Sequencing

The beta-globin gene (*HBB*) encodes a major subunit of hemoglobin and pathogenic variants in beta-globin, are associated with multiple phenotypes depending on whether one or both copies are altered, and on the type of variant (Table 4) (88). Disorders of hemoglobin, or hemoglobinopathies, are the most common monogenic disease with a combined carrier rate of 5%. In hemoglobinopathies, there is inadequate hemoglobin due to a reduction in synthesis (i.e., thalassemias) or due to structural changes (e.g., Sickle cell disease) in the encoded protein (89, 90).

**Table 4:** Common disorders related to the *HBB* gene.

**HBB disorder**	**Genotype**	**Phenotype**	**% HbA**
β-thalassemia minor	ββ^0^, ββ^+^ (i.e., carriers)	Asymptomatic or mild microcytic hypochromic anemia may be present.	92–95%
β-thalassemia intermedia	β^+^β^+^, β^+^β^0^ (typically with alpha gene deletion)	Later onset, microcytic hypochromic anemia, jaundice, hepatosplenomegaly, risk of iron overload.	10–30%
β-thalassemia major	β^0^β^0^, β^+^β^+^, β^+^β^0^	Onset within 2 years of life, severe microcytic hypochromic anemia, hepatosplenomegaly, failure to thrive.	0%
Sickle cell disease	HbS/HbS [homozygous for c.20A>T (p.Glu7Val)]	Onset in infancy, severe anemia, splenomegaly, jaundice, episodes of severe pain including swelling of hands and feet, stroke in childhood	Low to absent

*β, wildtype HBB locus; β^0^, pathogenic variant resulting in no production of HBB protein (e.g., deletion); β^+^, pathogenic variant resulting in reduced production of HBB protein (e.g., promoter variant)*.

While there are more than 300 unique pathogenic variants in *HBB* (89), >90% of patients have one of 15 variants (90, 91). Loss-of-function variants, referred to as β^0^ alleles, result in no protein production, while other variants such as missense or promoter variants result in reduced production of the HBB protein (β^+^). Large deletions in the promoter region of *HBB* are observed in certain populations and result in loss of HBB expression. The *HBB* gene is relatively small, and is composed of 3 exons which can be amplified and sequenced with only two amplicons. Due to its small size and the specific phenotype associated with *HBB* diseases, analysis of *HBB* alone is appropriate and is almost universally performed with Sanger sequencing to detect sequence variants, and MLPA to detect deletions (89).

#### Cystic Fibrosis: Targeted Mutation Analysis to NGS Panels

Cystic fibrosis (CF) is an autosomal recessive multi-systemic condition affecting the epithelia of the respiratory tract, exocrine pancreas, intestine, hepatobiliary system, and exocrine sweat glands (92). Clinical features include progressive obstruction of the lungs with worsening pulmonary disease, pancreatic insufficiency and malnutrition, recurrent sinusitis ad bronchitis and male infertility. CF is caused by mutations in the cystic fibrosis transmembrane conductance regulator (*CFTR*), which codes for a protein that functions as a chloride ion transporter. The 27 exon *CFTR* gene was identified as the CF disease gene by positional cloning based on linkage analysis in 1989, and was the first successful utilization of linkage analysis for disease gene identification (93).

Currently, over 1,000 distinct mutations have been identified, with a subset of relatively common mutations enriched in specific populations, and many rare mutations. There are several options for molecular diagnosis including (1) targeted genotyping analysis for common pathogenic variants, (2) Sanger sequencing of *CFTR* followed by deletion/duplication analysis if no sequence variants are found, or (3) sequencing of a multigene panel that includes *CFTR*, as well as other genes that may cause disorders on the differential diagnostic list for a patient with an uncertain diagnosis (92). Targeted analysis, which has been the first-line test for many years, must account for the individual's ethnicity, and provide a residual risk after a negative test [e.g., American College of Medical Genetics and Genomics recommended panel (94)]. On the other hand, sequencing of the entire gene with NGS, which has now become the most common technique for screening or diagnosis, can scan for all mutations in a single assay, reducing the need for reflex testing and improving the clinical sensitivity (92, 95, 96). When patients present with a broader differential, NGS panels including additional genes associated with primary ciliary dyskinesia, cholestasis, bronchiectasis, and/or congenital diarrhea, depending on the presenting features, are the most efficient route to a molecular diagnosis.

#### Noonan Spectrum: NGS Panel for a Distinctive but Genetically Heterogeneous Syndrome

Noonan spectrum is a group of disorders characterized by variable dysmorphic features, cardiac disease, short stature, developmental delay, and hematologic/oncologic disorders. Cardiac disease can manifest as structural heart disease, cardiac conduction defects, and cardiomyopathy. Patients have an ~10 times increased risk for childhood cancers, especially juvenile myelomonocytic leukemia and embryonal rhabdomyosarcoma, but risk varies by gene (97). Noonan syndrome is caused by activating mutations in a number of RAS pathway genes (most commonly *PTPN11, SOS1, RAF1*), and together with other similar, so-called RASopathy syndromes, over 20 genes have been implicated in this group of disorders which include Noonan syndrome with multiple lentigines, Cardiofacio cutaneous syndrome (associated with *BRAF, MAP2K1*, and *MAP2K2*), Costello syndrome (associated with *HRAS*), among others (98). Most forms are autosomal dominant, with *PTPN11* accounting for over half of Noonan syndrome patients, and many genes are associated with multiple RASopathies (98, 99).

While some genotype-phenotype associations exist, there is considerable phenotypic and genotypic overlap between RASopathies which can complicate diagnosis. Therefore, NGS gene panels are the most cost- and time-efficient path to diagnosis, and identifies an underlying genetic cause in 80% of cases (98, 100). Furthermore, these syndrome have important difference in terms of co-morbidities, and a genetic diagnosis aids in optimizing patient care (98, 99). For example, patients with Costello syndrome have a 40 times increased risk of malignancy, and require additional screening compared to other RASopathies (see section Genotype-First Approach for further discussion) (97, 101).

#### Hearing Loss: A Highly Heterogeneous Disorder Requiring Large Panel Testing

Hearing loss occurs in nearly 1/500 individuals and can have congenital, childhood, or adulthood onset (102–104). Greater than 50% of early onset hearing loss is caused by a single gene defect with the remainder due to other factors such as infection (103–106). Hearing loss can be isolated (non-syndromic) or associated with additional phenotypic features (e.g., Usher syndrome causing hearing loss, blindness and vestibular dysfunction and Jervell and Lange-Nielsen syndrome causing hearing loss and long QT syndrome). Hearing loss genetics is complicated by hundreds of associated genes that have autosomal recessive, autosomal dominant, X-linked and mitochondrial patterns of inheritance (107). Expert panel review of hearing loss genes reported in the literature has shown strong clinical validity for 142 genes, including 105 causing non-syndromic and 59 causing syndromic hearing loss (108). Thus, diagnostic testing requires comprehensive gene panels or ES to capture the diversity of genetic drivers giving rise to this phenotype (Figure 1C).

Despite this genetic heterogeneity, the majority of non-syndromic hearing loss is caused by two genes: gap junction protein β2 (*GJB2*) and stereocilin (*STRC*). Mutations in *GJB2* is the most frequent cause of severe-to-profound autosomal recessive non-syndromic hearing loss, occurring in up to 50% of these cases (109). Founder mutations c.35delG (European), c.167delT (Ashkenazi Jewish), and c.235delC (East Asian) have higher minor allele frequencies in their respective populations (1.6–0.6%), however the majority of pathogenic variants in *GJB2* are extremely rare and up to 70% of all *de novo* mutations in this gene are predicted to be pathogenic (110). Additionally, large deletions upstream of *GJB2* which includes the neighboring *GJB6* gene and impair expression of *GJB2* are prevalent in European populations (111). Thus, comprehensive testing for this gene requires gene sequencing (Sanger or NGS) and the ability to detect large CNVs upstream of the gene (e.g., SNP array). Mutations in *STRC* is the most common cause of mild-to-moderate autosomal recessive, non-syndromic hearing loss, causing up to 20% of these cases (112). *STRC* testing is complicated by the presence of a nearby pseudogene which is non-functional but contains nearly identical sequence information. To overcome this challenge, the *STRC* locus needs to be PCR amplified before sequencing analysis can be performed (113). Thus, a comprehensive diagnostic testing strategy is required for *GJB2, STRC*, and the >140 additional genes causing this phenotype (112, 114, 115). Using a comprehensive strategy that incorporates multiple laboratory methods ultimately yields a genetic diagnosis in 33–48% of hearing loss cases (112, 114, 115).

## Disorders Requiring Special Testing

### Repeat-Expansion Disease

Some disorders have long been known not to follow the rules of Mendelian inheritance, including Fragile X syndrome (FXS) and Spinal and bulbar muscular atrophy. The puzzle of their inheritance was solved in 1991, when cloning of the disease genes showed that these disorders were linked to repeat expansions in the corresponding genes (74, 75). Short tandem repeats (STRs), also known as microsatellites or simple sequence repeats, are tandemly repeated nucleotide motifs scattered throughout the human genome. Repeat expansion within STRs can happen due to genetic instability, and in a small subset of cases, can directly lead to human diseases, known as repeat-expansion disorders (Table 5).

**Table 5:** Examples of Repeat-expansion disorders.

**Disease**	**Inheritance**	**Gene**	**Type**	**Repeat motif**	**Normal range**	**Disease range**
Huntington disease	AD	*HTT*	Coding exon	CAG	≤26	>40
Spinal and bulbar muscular atrophy	X-linked	*AR*	Coding exon	CAG	≤34	≥38
Spinocerebellar ataxia 1	AD	*ATXN1*	Coding exon	CAG	6–35	≥39
Spinocerebellar ataxia 2	AD	*ATXN2*	Coding exon	CAG	≤31	≥33
Spinocerebellar ataxia 3	AD	*ATXN3*	Coding exon	CAG	12-44	~60–87
Spinocerebellar ataxia 6	AD	*CACNA1A*	Coding exon	CAG	≤18	20–33
Spinocerebellar ataxia 7	AD	*ATXN7*	Coding exon	CAG	4–35	37–460
Spinocerebellar ataxia 17	AD	*TBP*	Coding exon	CAG	25–40	≥49
Dentatorubral-pallidoluysian atrophy	AD	*ATN1*	Coding exon	CAG	6-35	48–93
Huntington disease-like 2	AD	*JPH3*	3′UTR, coding exon	CTG	6–28	40–60
Fragile X syndrome	X-linked	*FMR1*	5′UTR	CGG	6–54	200–1,000+
Fragile X-associated tremor/ataxia syndrome	X-linked	*FMR1*	5′UTR	CGG	6–54	55-200
Myotonic dystrophy 1	AD	*DMPK*	3′UTR	CTG	5–34	50–10,000
Myotonic dystrophy 2	AD	*CNBP*	Intron	CCTG	11–26	75–11,000
Friedreich ataxia	AR	*FXN*	Intron	GAA	5–33	66–1,300
Frontotemporal dementia and/or lateral sclerosis 1	AD	*C9orf72*	Intron	GGGGCC	<25	>60
Unverricht-Lundborg disease	AR	*CSTB*	Promoter	CCCCGCCCCGCG	2–3	≥30
Oculopharyngeal muscular dystrophy	AD	*PABPN1*	Coding exon	GCG	≤10	12–17

The repeat motifs vary in length, including trinucleotide repeats, such as CAG in Huntington's disease (HD), tetramer, pentamer, hexamer, and even dodeca-nucleotide repeats. The repeats can be located in the 5′ untranslated region (FXS), exon (HD), intron (Friedreich ataxia), or 3′ untranslated region (Myotonic dystrophy 1) of a gene (116–119). The length of pathogenic repeats vary between disorders, and are associated with multiple well-known mechanisms of pathogenicity, including loss of function (e.g., by hypermethylation of promoter, transcription interference or disruption of splicing); protein gain of function due to a polyglutamine/polyalanine tract expansion; and RNA gain of function to sequester the critical RNA splicing factors (119, 120). Despite the diversity of repeats and molecular mechanisms, the length of repeat expansion is usually positively correlated to the disease severity, but negatively correlated to the age of onset (121–125). One striking feature of these diseases is clinical anticipation, in which the disease tends to worsen in successive generations of a family as the repeat expansion extends. Indeed in repeat-expansion disorders, milder, or even distinct symptoms may be seen in individuals with so-called “permutation” alleles, that is, expanded alleles that are shorter than “full” mutations, but longer than normal alleles. Therefore, pediatric onset is usually associated with longer repeat expansions.

#### Molecular Genetic Testing

Diagnostic testing determines the number of repeats within the locus of interest. Additionally, in some cases such as FXS, methylation secondary to the aberrant repeat expansion is clinically important and must therefore be detected, for example with methylation-sensitive MLPA (see below). While traditional PCR and Southern blot analysis were once the standard diagnostic tests, they have now been replaced by repeat-primed PCR which is more efficient at identification of expanded repeats and at determination of the precise number of repeats. Of note, most disease-causing expansion alleles are longer than the read length of current NGS technologies, and repetitive sequences prevent the assembly of multiple reads into a consensus contig (126). As such, current NGS technologies are unable to accurately detect or size most tandem repeats; however, long-read sequencing is emerging to solve this problem (see section Long-Read Sequencing).

##### Repeat-primed PCR (RP PCR)

RP PCR uses three primers in a single PCR reaction, with two primers anchored outside the repeat to amplify the whole region, similar to a standard PCR amplification strategy. The third primer is a chimeric primer that anneals along the repetitive region to form multiple amplicons, which results in PCR products that differ in length by one repeat motif. Therefore, RP PCR increases the amount of full-length product for large repeat alleles, and allows the accurate detection of repeat numbers (127–129). However, there is a maximum number of repeats that can be quantified with RP PCR, and alleles greater than this can be detected but not quantified (e.g., 200 repeats for FXS).

#### Clinical Example

##### FXS, FXTAS, and FXPOF

FXS and related syndromes, Fragile X Tremor Ataxia Syndrome (FXTAS) and Fragile X Premature Ovarian Failure (FXPOF), are X-linked disorders caused by expansion of a CGG triplet in the 5′ untranslated region (the promoter) of the *FMR1* gene on the X chromosome. The *FMR1* gene encodes the FMRP protein, which is important for mRNA transport, translational regulation and synaptic plasticity (130). Fragile X is characterized by moderate intellectual disability, characteristic physical features (long face, large ears, prominent jaw and forehead, and enlarged testes in males after puberty) and behavioral abnormalities such as autism spectrum disorder, while FXTAS and FXPOF manifest later in life with progressive cerebellar ataxia and intention tremor, and ovarian insufficiency, respectively (131, 132).

Healthy individuals possess 5-44 CGG repeats (normal alleles) or 45–54 repeats (intermediate alleles) within the *FMR1* gene. Neither normal nor intermediate alleles cause disease, but a small percentage of intermediate alleles are unstable and may expand into the premutation range (55–200 repeats), which will lead to FXTAS (130, 131, 133). Both male and female carriers of a premutation can be affected by FXTAS with higher penetrance in males, while female have a 20% risk of FXPOF (127, 134). The premutation enhances *FMR1* transcription by shifting the transcription start site, and increases sequestration of RNA splicing proteins within the nucleus resulting in RNA-mediated toxicity (135, 136). A definite diagnosis of FXTAS requires the identification of a *FMR1* premutation as well as the neuroradiologic and clinical findings (132).

Premutation alleles can further expand to full-mutation alleles (>200 to over 1,000 repeats) which are often associated with aberrant hyper-methylation of the *FMR1* promoter and inhibition of *FMR1* transcription (137). Since it is the absence of FMR1 protein that leads to FXS, full-mutation alleles without hyper-methylation may not result in transcriptional silencing nor FXS (138). Female heterozygotes for the full-mutation may manifest with milder phenotypes of FXS. Less than 1% of FXS can be caused by *FMR1* deletion or single-nucleotide variants, which requires additional testing modalities such as those discussed in the previous sections (132, 139).

### Mitochondrial Diseases

Mitochondrial diseases are a rare group of disorders resulting from dysfunction of the mitochondria and are highly heterogeneous, both genetically and phenotypically. Mitochondria, the energy producing cellular organelles, are essential organelles present in almost all eukaryotic cells, and are involved in multiple cellular processes including calcium homeostasis, apoptosis, and most importantly oxidative phosphorylation for ATP production (140–143). Mitochondria are so important that mitochondrial defects often impact multiple organ systems, and the disorders can manifest in the neonatal phase, childhood or adulthood. Clinical variability is very common and the phenotypes do not always fit into a discrete syndrome. Therefore, diagnosis often relies on genetic testing (144).

The dual genetic control of mitochondrial function further complicates the mechanisms of mitochondrial diseases, as the 37 genes in the mitochondrial genome (mtDNA) and over 1,500 nuclear genes control mitochondrial function together (145–147). Consequently, mitochondrial diseases can have autosomal or X-linked inheritance for nuclear gene mutations, or maternal inheritance for mtDNA mutations. Furthermore, there are many mitochondria present in each cell and the clinical presentation of mitochondrial diseases is, in part, a function of the frequency of the mutant mtDNA sequence in each cell (i.e., heteroplasmy). Disease manifestation generally requires heteroplasmy levels above a certain threshold, and increasing levels of heteroplasmy are often associated with increasing clinical severity (144, 148).

#### Molecular Genetic Testing

Testing for mitochondrial disease is complicated not only by the need to assay both nuclear and mitochondrial genomes, but also because there are hundreds of genes implicated with mitochondrial disease, and for mtDNA mutations, tissue-specific heteroplasmy complicates interpretation.

Historically, targeted mutation analysis for common, known pathogenic variants was performed by one of several hybridization-based techniques which were limited in the scale and scope of testing. Sanger sequencing of the entire mitochondrial genome allowed for an unbiased assessment of the mtDNA, but required ancillary testing to assess heteroplasmy of an identified variant. Large deletions were typically identified through Southern blot analysis of DNA extracted from muscle samples, but sensitivity of lower heteroplasmy levels is limited. The emergence of NGS has enabled simultaneous interrogation of both point mutations and deletions in nuclear genes, and in mitochondrial genes when combined with long range PCR amplification of mtDNA (Figure 1D). The additional advantage of NGS is the quantitative nature of the sequencing, providing an estimate of heteroplasmy for a given variant.

##### Leigh syndrome

Leigh syndrome, the most common mitochondrial disease in childhood, is a progressive neurodegenerative disorder with typical onset before 2 years of age. Patients show brainstem and/or basal ganglia dysfunction, elevated serum or cerebrospinal fluid lactate, and neurological features including developmental delay and regression, hypotonia, spasticity and peripheral neuropathy (149). Episodic progression of the disease typically results in death before 3 years of age due to respiratory or cardiac failure. Over 75 mitochondrial or nuclear genes are linked to LS, and almost one-third of these genes are related to complex I function. Mutations in mitochondrial genes contribute to ~20% of LS, with the mtDNA mutations m.8993T>G and m.8993T>C in *MT-ATP6* underlying ~10%. Most of the mutations in mitochondrial genes require more than 90% heteroplasmy to cause the LS symptoms. Rarely, LS can also result from a large-scale mtDNA deletion (149). Currently, the molecular diagnosis of LS is a two-pronged NGS approach with large panels or ES for nuclear genes and long-range PCR amplification of mtDNA followed by NGS (Figure 1D).

##### Mitochondrial DNA deletion syndromes

Large mitochondrial DNA deletions cause a spectrum of diseases including three major groups according the distribution of disease manifestation (150). Kearns-Sayre syndrome (KSS) with mtDNA deletion present in all tissues, is a progressive multisystem disorder with onset before the age of 20. KSS patients have progressive external ophthalmoplegia and pigmentary retinopathy, as well as cardiac conduction defects or cerebellar ataxia. Pearson syndrome with mtDNA deletions that are abundant in blood leukocytes, is characterized by sideroblastic anemia. Progressive external ophthalmoplegia (PEO) with mtDNA deletion confined to skeletal muscle, can manifest with ptosis, impaired eye movements, oropharyngeal weakness and variably severe proximal limb weakness. Almost all mitochondrial DNA deletion syndromes occur *de novo*. Approximately 90% of KSS cases arise from a *de novo* large scale 1.1–10 Kb deletion of mtDNA that includes ~12 genes. The most common deletion known as the “common 4,977 bp deletion” accounts for over one third of total cases. NGS on the products of long-range PCR targeting mtDNA can reveal one or more mtDNA deletions with the exact breakpoints, and also estimate the heteroplasmy of the deletion.

### Imprinting Disorders

A small fraction of genes are marked during gametogenesis as being transmitted through the maternal or paternal gamete, and these genes are only expressed if transmitted either maternally or paternally. In other words, they are subject to monoallelic, parent-of origin specific expression (151). Imprinting is established during gamete formation and many imprinted genes are important for embryonic development and early postnatal growth, and therefore, imprinting disorders tend to share common features including aberrant fetal or postnatal growth, abnormalities in glucose regulation, abnormal neurologic behaviors or mental deficits and abnormal gonadal maturation. At least 150 human imprinted genes were identified by 2015, and most exist in clusters (152, 153). The imprinted expression pattern of these gene clusters are epigenetically controlled by methylation at *cis*-acting discrete elements known as imprinting control regions (154).

Perturbed expression of imprinted genes leads to congenital disorders of imprinting, and there are four major categories of mutations that will cause disease. These four categories are (1) chromosomal rearrangements such as deletions, which can result in lack of expression of the corresponding imprinted genes; (2) uniparental disomy (UPD) in which both copies of a chromosome, or chromosomal segment, are inherited from one parent; (3) imprinting defects, which are caused by aberrant methylation of the imprinting control regions or small deletions involving the imprinting center; and (4) intragenic mutations in imprinted genes that disrupt the normal expression or function of those genes. Intragenic mutations are rare overall, but relatively common when imprinting disorders are inherited (155).

#### Molecular Genetic Testing

Multiple techniques have been developed to interrogate one or multiple disease mechanisms, but methylation-specific PCR (MS-PCR) and methylation-specific MLPA (MS-MLPA) are the gold-standard techniques for imprinting disorders due to their ability to identify abnormal DNA methylation patterns irrespective of the underlying disease mechanism (Figure 1E). However, the underlying mechanism is important for genetic counseling issues such as recurrence risk, and additional techniques are needed delineate it. FISH, CMA, or MLPA can be performed to detect a deletion; SNP arrays or STR analysis can be used to evaluate UPD; MLPA can be used to detect deletions of imprinting control regions; while NGS or Sanger sequencing can detect intragenic mutations in the imprinted genes. Depending on the disease, each mechanisms accounts for varying proportions of patients, and the order of reflex testing is adjusted accordingly.

##### MS-PCR

The critical component of MS-PCR is bisulfite treatment of DNA prior to amplification. Sodium bisulfite treatment of DNA converts all unmethylated cytosines to uracil, which can reveal the methylation differences between paternal and maternal alleles. Two pairs of primers are designed based on the converted maternal and paternal sequences, respectively. The PCR amplification products from maternal and paternal DNA can be separated in electrophoresis because of their different sizes (156). Therefore, MS-PCR can detect loss of maternal or paternal alleles caused by deletion, UPD or imprinting defect, but additional testing is required to reveal the underlying mechanism of the imprinting defect.

##### MS-MLPA

MS-MLPA uses methylation-sensitive endonucleases which digest unmethylated DNA and prevent probe ligation and amplification. Therefore, only methylated alleles are visualized and parent-of-origin can be determined based on test design. MS-MLPA is typically performed in parallel with regular MLPA to detect any underlying CNVs in the region of interest. Therefore, the combination of MS-MLPA and standard MLPA can identify the loss of a maternal or paternal allele and an underlying deletion or ID, but additional testing is required to detect UPD or sequence variants (157).

#### Clinical Examples

##### Prader-Willi syndrome (PWS) and Angelman syndrome (AS)

PWS and AS result from imprinting abnormalities at 15q11.2-q13 and illustrate how disruption of imprinting patterns on either the maternal or paternal allele result in distinct diseases. The imprinting control regions within 15q11.2-q13 is normally unmethylated on the paternal allele, and methylated on the maternal allele. Therefore, in MS-PCR and MS-MLPA, loss of the maternal PCR product suggests AS, while loss of paternal PCR product indicates PWS. Overall MS-PCR and MS-MLPA can diagnose ~99% of PWS and ~80% of AS, and 11% of AS patients are diagnosed by sequencing of *UBE3A* (158, 159).

PWS is clinically characterized by severe hypotonia and feeding difficulty in early infancy, obesity caused by hyperphagia in later infancy or early childhood, developmental delay, intellectual disability, distinctive behavioral abnormalities, hypogonadism, and characteristic facial features (159, 160). PWS is caused by genomic or epigenetic changes that result in loss of expression of at least 13 paternally expressed genes on chromosome 15q11.2-q13, and deficiency of *SNORD116* is thought to be responsible for the key characteristics of PWS (160). There are three major mechanisms leading to PWS. The most common, accounting for 65–75% of patients, is a paternal deletion of 15q11.2-q13, most commonly one of two recurrent deletions (type I: ~6 Mb and type II: ~5.3 Mb). Maternal UPD of chromosome 15 is identified in 20–30% of PWS patients, in which ICRs of both alleles are methylated so that normally paternally expressed genes are silenced on both alleles. An imprinting defect accounts for 2.5% of PWS patients (159).

AS is characterized by severe developmental delay or intellectual disability, severe speech impairment, movement, or balance problems, and a unique behavioral phenotype with a happy (although sometimes inappropriate) demeanor (158, 161–163). Clinical manifestations are usually apparent after 1 year of age. In contrast to PWS, AS is caused by the genomic or epigenetic changes that lead to loss of expression of the maternally expressed genes within the chromosome 15q11.2q13 imprinted region. The fundamental features of AS are believed to result from deficiency of *UBE3A* expression in the neurons, where the paternally expressed *SNURF-SNRPN* transcript serves as an antisense RNA for the *UBE3A* gene (158, 162, 163). AS can be caused by deletion of the 15q11.2-q13 critical region on the maternal allele (65–75%), paternal UPD (3–7%), imprinting defect (~3%), and maternal *UBE3A* pathogenic variants (11%).

## Impact on Clinical Care

### The Importance of Genetic Testing

The evolution of genetic testing has had a tremendous impact on the clinical care of patients. As our diagnostic capabilities continue to expand, our ability to provide a diagnosis for our patients is rapidly increasing. For many families, simply having an answer for the cause of their child's medical issues can be very powerful. At times, answering the “why” is a family's primary motivation for pursing genetic testing. Having a name for the condition allows them to find a peer group with parents of similarly affected children with whom they find shared experiences. Additionally, many families unnecessarily harbor a great deal of personal guilt about their child's condition, having the belief that they somehow caused their child's medical issues. Being able to provide a concrete genetic answer can alleviate that guilt and allows the family some closure.

A specific genetic diagnosis provides a general prognostication for a patient's future. This can be particularly impactful for infants and small children who may have yet to manifest some of the comorbidities of the condition. It allows for realistic expectations to be set with the parents and allows for directed care with condition-specific recommendations. The intention is to not set up a self-fulfilling prophecy, but rather ensure that all of the necessary support is in place to allow the individual to achieve to their maximum potential.

Providing a genetic diagnosis can also significantly impact family planning. Many couples are hesitant to expand their family without knowing the underlying cause of their child's condition. Once the genetic etiology is known, that information can be used to make an educated decision about if and how they decide to have more children. If they so choose, a specific gene mutation can be used for pregnancy screening and/or *in vitro* fertilization.

### Genotype-First Approach

As genetic testing has become more readily available and expansive, the practice of clinical genetics has advanced as well. Recently, there has been a trend toward the early genotyping of patients. This has multiple benefits in regards to patient care. For conditions that are highly heterogenous, NGS panels and ES allow for the simultaneous analysis of hundreds or thousands of genes, resulting in higher diagnostic rate in those groups of disorders. Epilepsy is one such example in which there is an ever-expanding list of genes that are known to be causative in both syndrome and non-syndromic phenotypes. Helbig et al. (164) showed that ES was not only an efficient means to the identification of known disease-causing genes in patients with epilepsy, but also allowed for the identification of novel candidate genes. In their cohort, the diagnostic rate varied by phenotype, with epileptic encephalopathy patients having the highest yield.

In the recent past, CMA have been recommended as the first line test for individuals with neurodevelopmental disorders with a diagnostic yield of 15–20% (19, 165, 166). In a recent meta-analysis by Srivastava et al. (76) a review of 30 articles with data on the yield of ES in neurodevelopmental disorders showed that the test was diagnostic in 36% percent overall, with 31% in isolated neurodevelopmental disorders and 53% in neurodevelopmental disorders with additional features. Based on that data, given the higher diagnostic rate in ES when compared to CMA, they proposed that ES be considered as a first line test in that group of disorders. With time, it is anticipated that ES will become a much more widely used first line tool for the evaluation of many varied phenotypes.

Utilizing a genotype-first approach allows for the avoidance of unnecessary evaluations and diagnostic studies, and permits condition-specific care. For example, sensorineural hearing loss is a highly heterogeneous condition (see section Hearing Loss: A Highly Heterogeneous Disorder Requiring Large Panel Testing) in which ~30% of individuals have a syndromic form of hearing loss that have a variety of known comorbidities (167). Due to the variability in presentation and age of onset, it has been proposed that individuals with sensorineural hearing loss should undergo screening evaluations for comorbidities in order to guide genetic testing (168). Recommended evaluations include an ophthalmology evaluation to assess for vision differences seen in Usher syndrome, renal ultrasound to look for structural differences seen in Branchio-oto-renal syndrome, cardiac evaluation to evaluate for cardiac conduction defects associated with Jervell and Lange-Nielson syndrome, and temporal bone MRI to assess for the inner ear anomalies seen in Pendred syndrome. However, as the understanding of the genetic etiology of sensorineural hearing loss improves and as testing becomes more available, many of these evaluations become unnecessary if the genotype is able to be established soon after the hearing loss is detected via genetic testing (104).

At times, evaluation of a suspected genetic diagnosis may require painful or invasive procedures for confirmation of a suspected diagnosis. Osteogenesis imperfecta is a group of disorders characterized by bone fragility due to defects in type 1 collagen resulting in fracturing with minimal trauma. Prior to the expansion of our knowledge of the underlying genetic etiologies for the condition, the diagnosis often relied on clinical criteria and biochemical analysis via skin fibroblasts (169, 170). Now, however, biochemical analysis is much more rarely utilized as we rely on molecular studies for diagnosis. Likewise, neuromuscular conditions are a highly heterogeneous group of disorders that include neuropathies, myopathies, motor neuron disease, and neuromuscular junction disorders. Given the expansive differential for these conditions, clinicians have traditionally utilized such procedures as muscle biopsies and electromyography (EMG) as a diagnostic means in order to narrow their differential prior to proceeding with targeted testing (171, 172). As large scale genomic testing becomes available, however, it is predicted that those painful procedures may be able to be avoided as first line evaluations.

Perhaps most importantly, establishing a genetic diagnosis allows for condition-directed care. Identifying an underlying genetic etiology for an individual allows for appropriate specialty referrals to assess for associated comorbidities. For some conditions, a particular diagnosis may necessitate dietary or medication changes, alter screening recommendations, or impact an individual's ability to qualify or benefit from a transplant procedure.

For example, in Noonan spectrum disorders (see section Noonan Spectrum: NGS Panel for a Distinctive But Genetically Heterogeneous Syndrome), the type of cardiac risk varies depending on the underlying genetic etiology (101, 173), with pulmonary stenosis being more common in *PTPN11* disease and hypertrophic cardiomyopathy present in 95% of individuals with *RAF1* mutation. Additionally, individuals with mutations in *PTPN11*, particularly those with germline pathogenic variants in codons 61, 71, 72, and 76, are at risk for juvenile myelomonocytic leukemia (JMML), and patients with HRAS mutations have a significantly increased risk of malignancy even compared to other RASopathies (101, 174). Therefore, knowing the genotype of the patient ensures that proper screenings are in place. Given the phenotypic variability of Noonan spectrum disorder, and the importance of genotype identification, targeted panel testing is recommended.

### Counseling and Ethical Considerations

Along with genetic testing comes a myriad of ethical considerations. Vital to the practice of clinical genetics is maintaining an individual's autonomy, particularly in regards to the use of genetic testing. Prior to proceeding with any testing, it is important that informed consent be obtained, ideally by an individual with a background in clinical genetics. As part of the informed consent process, pretest counseling should include a discussion about potential test results or expected outcomes, possible benefits and limitations of testing, and the possibility of incidental or secondary findings (175).

Secondary findings are a particularly important and challenging point of discussion as individuals are typically asymptomatic in that regard. In the context of ES and GS, currently, the American College of Medical Genetics recommends that an individual should be offered analysis of a minimum of 59 genes that are considered medically actionable in that they would convey a change in medical management if identified (see also section Variant Classification) (86). Included in that list are cancer predisposition genes such as *BRCA1* and *BRCA2* as well as genes that are associated with risk of cardiomyopathy. Given the impact that these findings have on an individual's care, it is important that this testing be thoroughly discussed prior to proceeding.

As part of the informed consent process for genomic testing, it is important for individuals to understand the implications that genetic test results could potentially have on employment and insurance eligibility. In 2008, the Genetic Information Non-discrimination Act (GINA) was passed into law (176). GINA prohibits health insurers form using genetic information to discriminate against individuals in regards to their eligibility and coverage. It also prevents employers from using that information to make employment decisions including hiring, pay, and firing. However, there are caveats to the policy and some people are not protected by this law including, individuals who work for employers with fewer than 15 employees, individuals with Federal or military insurance, and people with additional insurance policies including long-term and life insurance. It is important that these protections and limitations are discussed and understood prior to proceeding with testing. This is particularly important when discussing pre-symptomatic testing.

A particularly challenging ethical area is genetic testing in children. Genetic testing for children who are symptomatic of a genetic condition is typically considered acceptable with appropriate pre and post-test counseling (177). When applicable, it is important to involve the child or adolescent in the discussion and obtained their assent prior to proceeding with testing (178). However, broad carrier screening and testing for adult onset conditions are not recommended in the pediatric population as to maintain autonomy for that individual to make that decision for themselves in adulthood (177, 179).

As large-scale genomic testing becomes more commonplace, we will undoubtedly continue to encounter an ever-expanding list of ethical concerns that will need to be discussed. It will be important to readdress these issues as the field evolves.

## Emerging Technologies

The diversity of genetic diagnostic testing methods reflects the diversity of genomic alterations that cause Mendelian disorders. As NGS-based assays have transitioned from being used primarily in research settings to becoming established clinical assays, it remains to be seen whether newer techniques can become standard in clinical practice. The technologies listed below represented techniques recently adopted or on the cusp of adoption in clinical laboratories. One promising techniques which continues to be evaluated is optical mapping which is a non-sequencing genome assembly assay. Optical mapping is promising for the resolution of structural variants (180), repeat expansion (181), and may dramatically impact cancer cytogenetics by offering one assay to replace karyotype, FISH and CMA (182). Indeed, the trajectory of pediatric genetic testing appears focused on streamlining approaches to limit the number of tests required to identify any disease causing mutation to save both time and reduce costs. Whether a “one test fits all” approach is practical or even desirable has yet to be clearly demonstrated from a technical, patient care, or cost-benefit perspective. Nonetheless, leading the way for a single, comprehensive genomic diagnostic test for rare disorders is clinical GS.

### Clinical Genome Sequencing

In the next 5–10 years, GS is poised to supplant ES as the most comprehensive clinical genomic diagnostic test. Technical advantages of GS over ES include (1) more even coverage across exons, (2) coverage of non-coding regions of the genome (e.g., intergenic, promoter, UTR, and intron), (3) comparable CNV detection to CMA (including mapping breakpoints), (4) the ability to detect structural rearrangements such as balanced translocations and inversions, and (5) faster and cheaper library preparation (183–186). The major limitations of GS compared to ES is the bottleneck created after libraries are made since (1) fewer patient samples can be sequenced per run and (2) the bioinformatic processing of the increased amount of data is more computationally intensive and requires increased storage space. The additional work and storage needs contribute to GS being at least twice as expensive to perform (187). Despite the current logistical challenges of implementing GS for all genetics patients, numerous studies have shown that GS can be done rapidly in the setting of acutely ill, neonatal and pediatric intensive care unit patients (72, 73, 188–191). These studies performed in a research and clinical laboratory setting has provided diagnoses primarily for patients with multiple congenital anomalies and neurological phenotypes (75, 189). Guidelines for a “rapid genome” are somewhat ambiguous as some studies show a turnaround time of 28 days (189) while others have results closer to 24 h (73). Diagnostic yield also varies between studies and suffers from case-selection bias; nonetheless, most studies found diagnoses in 28–43% of patients (72, 73, 189). These successful efforts show GS is gaining significant traction for immediate testing of critically ill children with suspected Mendelian disorders and we anticipate that more studies will demonstrate the efficacy of this approach in the clinical laboratory.

The wider implementation of GS for non-critically ill patients is likely to evolve more slowly as early adopters of this technology will need to demonstrate its increased diagnostic utility compared to ES and/or evidence that advancements in sequencing and computational technology has made it cost effective. Meta-analysis of the median diagnostic yield from 37 studies, comprising 20,068 children found a diagnostic rate of 41% (95% CI 34–48) for GS and 36% (95% CI 33–40) for ES, which are both significantly better than CMA (diagnostic yield of 10%, 95% CI 8–12), but not significantly different from each other (75). Comparable diagnostic yield between GS and ES was further replicated in two additional studies not included in this meta-analysis (192, 193). The inability of GS to show a marked increase in diagnoses is likely due to restricting variant analysis to coding regions. Analyzing non-coding variants is one possible way to gain new diagnoses that would otherwise be missed by ES, however analysis of these variants poses multiple challenges. For instance, a proband with a *de novo*, deep-intronic or promoter variant that has not been previously reported as disease causing in a gene of interest, while interesting, is unlikely to be diagnostic since the consequence of this mutation needs functional assessment of the impacted transcript (84).

Studies on the genetics of autism and related neurodevelopmental disorders could push GS into new territory with recent work suggesting an excess of *de novo* mutations in enhancer elements of neurodevelopmental genes in affected individuals (194–198) and may account for 1.0–2.8% of negative exomes (197). However, interpreting the additive role of *de novo* and common population variants across multiple genes and non-coding elements poses significant analytical challenges. Although oligogenic inheritance models have been hypothesized for autism and other disorders (199–201), this is not uniformly supported (202) and the data does not meet standards for clinical validity for diagnostics at this time. Lastly, while it is enticing to think that genome sequencing can act as a catch-all diagnostic test, certain mutation types such as repeat expansions and regions of high homology (e.g., segmental duplications and pseudogenes), remain very difficult to assay by short-read sequencing (190). Altogether, clinical GS is emerging as a frontline diagnostic test but is likely a few years away from usurping ES as the premiere testing method for rare disorders.

### Long-Read Sequencing

Most next-generation sequencing protocols used in clinical laboratories start with the controlled fragmentation of the human genome to create 200–500 bp DNA fragments. Following library preparation these fragments are sequenced with short-read lengths of 100–250 bp by Illumina-based platforms. Although short-read lengths enable highly accurate identification of SNVs and small indels, this approach has difficulty with other types of genetic variants like nucleotide repeat expansions, distinguishing regions of high homology, structural variants (including copy neutral) and phasing pathogenic alleles. To overcome these challenges, long-read sequencing technologies have been developed to create read lengths of >10,000 bp (203, 204). Having long reads allows for sequencing across challenging genomic regions and may even help unmask novel damaging variants and discover new disease genes (205, 206). Two emerging technologies leading the way in this field include single molecule real-time (SMRT) sequencing (Pacific Biosciences) and measuring electric currents as nucleic acids pass through a protein nanopore (Oxford Nanopore Technologies) (203, 204). Clinical laboratories have been reluctant to adopt these technologies in part because they initially had high costs, high error rates for base pair calling (PacBio ~15%, Nanopore 5–25%), and unestablished clinical validity (207–209). However, newer versions of these sequencing platforms have seen improvements in cost and performance such that a case can now be made for laboratories to explore their utility for diagnostic testing. In a research setting this technology can identify clinically relevant structural variants (210–213), sequence entire nucleotide repeat expansions found in genes like *HTT, FMR1*, and *ATXN10* (214–216), bypass pseudogenes affecting *IKBKG* and *CYP2D6* (217, 218), and phase disease alleles (219). Although the studies outlined above provide evidence that challenging genomic regions can be reliably analyzed with long-read technology, an initial long-range PCR to enrich for the target region was often performed limiting the scalability and clinical utility of these methods. A potential future clinical application built upon continued improvement in technology and sequencing throughput would be performing long-range sequencing across the whole genome and doing *de novo* assembly which would yield fully phased patient specific genomes generated independent of alignment to a reference sequence (220, 221). *De novo* assembly of long-read patient genomes represents the future of this emerging technology allowing detection of all normal and pathogenic genomic variation.

### RNA-Sequencing and Methylation Pattern Analysis

Sequencing of messenger RNA (mRNA) can provide unique information compared to DNA sequencing as it (1) allows interrogation of the results of splicing, (2) detects allele specific expression, and (3) allows examination of mRNA abundance, in addition to identifying germline variants found in coding regions of expressed transcripts (222–225). Thus, RNA-seq can be seen as a hybrid test that assesses abnormal expression and splicing and capable of detecting pathogenic coding variants. Improving methods to detect abnormal splice events is important to note since it is estimated that nearly 10% of all disease causing variants disrupt splicing (226) and variants impacting canonical splice sites at intron-exon boundaries are categorically defined as null variants by ACMG guidelines (84, 227). Detection of abnormal splicing by RNA-seq could therefore emerge as a critical reflexive diagnostic test for negative exomes and genomes (222).

An ongoing challenge associated with implementing clinical RNA-seq is deciding which patient tissue to assay, since tissue restricted transcript expression and alternative splicing limit the total number of genes that can be reliably tested for abnormalities. Fortunately, databases such as the Genotype-Tissue Expression (GTEx) project, which provides the expression level and transcriptional diversity of 53 non-diseased tissues from nearly 1,000 adults (228), can be used as a reference for the expected transcriptional state of biopsied tissue. Tissues from patients that are accessible for RNA-seq testing include fibroblasts derived from skin biopsy (229), muscle biopsy (222, 223), whole blood (224), and B lymphoblastoid cell lines made from Epstein-Barr virus transduced whole blood. RNA-seq has so far been performed on muscle biopsies and fibroblasts for a restricted set of neuromuscular and mitochondrial diseases, respectively (222, 223, 229). RNA-seq on a cohort of 25 neuromuscular disorder patients with negative exome and/or panel testing had a diagnostic rate of 36% (222) and in 48 mitochondriopathy patients a diagnosis was reached in 10% of cases (229). RNA-seq testing of whole blood in 94 patients with a broad set of phenotypes resulted in a diagnostic rate of 7.5% (224). These studies detected multiple pathogenic mRNA mechanisms causing premature termination, including exon skipping, activation of cryptic donor and acceptor splice sites, and incorporation of *de novo* exons. From these studies, RNA-seq appears to have the greatest benefit when affected tissue is assayed (e.g., muscle for patients with neuromuscular phenotypes) in patients that have exhausted all current standard of care DNA sequencing tests (222, 223). Another interesting challenge that emerges with clinical RNA-seq of mature mRNA is the possibility of detecting an abnormal transcript (e.g., defective splice product) but not identifying the underlying genomic variant causing the event (e.g., deep intronic mutation). Thus, genetics professionals will need to establish appropriate guidelines for how to interpret pathogenicity of abnormal splice products without knowing the causative genomic variant. Perhaps a future of GS paired with RNA-seq will provide the greatest benefit to patients since suspected pathogenic non-coding variants detected by GS can be interpreted concurrently for their functional impact by RNA-seq.

An interesting new approach to determine whether or not someone is affected with a Mendelian disorder is to assay changes in DNA methylation from whole blood. This approach is based on recent studies showing mutations in genes functioning as epigenetic and transcriptional regulators frequently result in neurodevelopmental disorders (230), and as a result of their molecular functions drive unique DNA methylation signatures that mirror the gene containing the primary mutation (231–233). Thus, an individual with a mutation in the histone methyltransferase *KMT2D* causing Kabuki syndrome results in a unique DNA methylation profile compared to someone with a mutation in the SWI/SNF complex gene *ARID1B* causing Coffin-Siris syndrome (231, 232). This diagnostic testing approach has made the jump to clinical testing and will be especially useful when variants of uncertain significance are detected in chromatin remodeling genes or for reflex testing from negative exomes. Limitations of this approach are mainly the restricted number disorders that transmit unique and reproducible methylation signatures in blood. Additionally, similar to RNA-seq, this method reveals functional consequences instead of causative genomic variants, thus scenarios where novel signatures are detected would likely be non-diagnostic until the culprit genomic variant is found.

## Discussion

Genomic diagnostic testing for pediatric disorders has transformed from low resolution (e.g., karyotype) and single locus (e.g., Sanger, FISH, PCR) analyses to high-throughput and high resolution genome-wide testing (e.g., CMA, ES, GS), with an-ever diminishing distinction between cytogenetic and molecular genetics (Table 6). These technical advances have transformed how rare diseases are diagnosed and treated, and has massively broadened the spectrum of genes causing rare Mendelian disorders. However, the widespread adoption of these methods has led to challenges in how to rapidly and accurately interpret the staggering number of single nucleotide and copy number variants that exist in the human genome. The interpretation of and counseling for the many variants of uncertain significance remains an enormous challenge. These uncertain diagnoses can become especially problematic in disorders such as hearing loss which can have mild phenotypes indistinguishable from environmental insults and emerge progressively and later in life. Aside from variants that are strictly rare and therefore of unknown impact, there is also the problem of the limited information available for many genes. Some disease-causing genes have only been reported in a few patients and thus an incomplete phenotypic spectrum. The challenge on this front is therefore whether there is convincing overlap between what is known about the gene and the patient's phenotypes, highlighting the importance of the partnership between clinical and laboratory geneticists. Education and counseling for both non-genetics clinicians and patients on the significance of secondary findings is also a current challenge, and highlights the need for appropriate informed consent prior to undertaking genetic studies. Currently, the genomics field is moving toward improved diagnostic rates through utilization of new technologies and the increasing use of genome sequencing, to limit the number of tests required for diagnosis and decrease turn-around times. As genomic knowledge increases, we anticipate that tests will become more definitive and will provide answers for an increasing number of patients. The education of non-geneticists and patients remains a key challenge and we look forward to the increased utilization of genomic information in medical management.

**Table 6:** Key points from review.

- Technical advances have driven changes in genomic diagnostics - CMA enables genome-wide detection of CNVs, including more efficient detection of atypical CNVs associated with recurrent CNV syndromes - NGS enables cost-efficient, high-throughput sequencing - Diagnosis often requires multiple technologies, either to detect different types of genetic variants, or to narrow the differential diagnosis - Panel-based testing has largely supplanted individual gene tests - Certain conditions require specialized testing and are not covered in exome sequencing, such as repeat-expansion disorders and imprinting disorders - Clinical care is moving toward a genotype-first approach - Interpretation of genetic data is imperfect; variants of unknown significance are common and pose challenges for genetic counseling - High-throughput evaluation can identify incidental and secondary variants outside the indication for testing - Pre- and post-test genetic counseling is critical - Clinical genome sequencing is emerging and is expected to further change genetic diagnostics as sequencing costs continue to decrease

## Author Contributions

All authors listed have made a substantial, direct and intellectual contribution to the work, and approved it for publication.

## Conflict of Interest

The authors declare that the research was conducted in the absence of any commercial or financial relationships that could be construed as a potential conflict of interest.
